# N6-Methyladenosine Regulates the Expression and Secretion of TGFβ1 to Affect the Epithelial–Mesenchymal Transition of Cancer Cells

**DOI:** 10.3390/cells9020296

**Published:** 2020-01-25

**Authors:** Jiexin Li, Feng Chen, Yanxi Peng, Ziyan Lv, Xinyao Lin, Zhuojia Chen, Hongsheng Wang

**Affiliations:** 1 Department of Microbial and Biochemical Pharmacy, School of Pharmaceutical Sciences, Sun Yat-sen University, Guangzhou 510006, China; lijiexin3@mail.sysu.edu.cn (J.L.); chenf57@mail2.sysu.edu.cn (F.C.); yx117pengyanxi@163.com (Y.P.); lvzy5@mail2.sysu.edu.cn (Z.L.); linxy49@mail2.sysu.edu.cn (X.L.); 2 Sun Yat-sen University Cancer Center, State Key Laboratory of Oncology in South China, Collaborative Innovation Center for Cancer Medicine, Guangzhou 510060, China

**Keywords:** m^6^A, TGFβ1, EMT

## Abstract

N6-methyladenosine (m^6^A) is the most abundant modification on eukaryotic mRNA, which regulates all steps of the mRNA life cycle. An increasing number of studies have shown that m^6^A methylation plays essential roles in tumor development. However, the relationship between m^6^A and the progression of cancers remains to be explored. Here, we reported that transforming growth factor-β (TGFβ1)-induced epithelial–mesenchymal transition (EMT) was inhibited in methyltransferase-like 3 (METTL3) knockdown (Mettl3^Mut/−^) cells. The expression of TGFβ1 was up-regulated, while self-stimulated expression of TGFβ1 was suppressed in Mettl3^Mut/−^ cells. We further revealed that m^6^A promoted TGFB1 mRNA decay, but impaired TGFB1 translation progress. Besides this, the autocrine of TGFβ1 was disrupted in Mettl3^Mut/−^ cells via interrupting TGFβ1 dimer formation. Lastly, we found that Snail, which was down-regulated in Mettl3^Mut/−^ cells, was a key factor responding to TGFβ1-induced EMT. Together, our research demonstrated that m^6^A performed multi-functional roles in TGFβ1 expression and EMT modulation, suggesting the critical roles of m^6^A in cancer progression regulation.

## 1. Introduction

In eukaryotes, gene expression is regulated by transcriptional and post-transcriptional processes. Among the over 100 modifications in mRNA, N6-methyladenosine (m^6^A) is one of the most abundant internal modifications [1,2,3]. This methylation process is catalyzed by “writer” methyltransferases, including methyltransferase-like 3 and 14 (METTL3 and METTL14) and other cofactors, such as Wilm’ tumor-1-associated protein (WTAP). m^6^A-modified mRNA is recognized by distinct “reader” proteins containing the YT521-B homology (YTH) domain, such as cytoplasmic proteins YTHDF1, YTHDF2, YTHDF3, and nuclear proteins YTHDC1 and YTHDC2. “Eraser” demethylases, alkylation repair homolog protein 5 (ALKBH5), and the fat mass and obesity-associated protein (FTO) are responsible for maintaining the balance of m^6^A methylation and demethylation in mammalian cells [4,5].

A growing body of work provides evidence showing that m^6^A mRNA modification acts as a regulator of the mRNA life cycle, including pre-mRNA splicing [6,7,8], nucleo-cytoplasmic export [5,9,10], mRNA decay [11,12], and mRNA translation [13,14]. The METTL3–METTL14 heterodimeric complex generates m^6^A on mRNA, where METTL3 contributes the catalytic residues and METTL14 provides structural supports for METTL3 [15,16,17]. Acting as the executor of m^6^A modification, METTL3 plays crucial roles in various biological processes, including tumor development [18,19]. For instance, METTL3 is necessary for the development and maintenance of mouse and human myeloid leukemia [20]. Our recent study indicated that METTL3 regulated the epithelial–mesenchymal transition (EMT) of cancer cells via Snail translation [21]. Although associations between m^6^A methylation and tumorigenesis, especially EMT process, have arisen for the last decade, the detailed mechanisms remained to be elucidated.

EMT of cancer can be induced by a plethora of signaling pathways, and transforming growth factor β (TGFβ) is the prominent EMT inducer in cancer cells [22]. TGFβ, which contains three isoforms, TGFβ1, TGFβ2, and TGFβ3, is synthesized as a pro-protein monomer. During the maturation, the TGFβ dimer forms a complex with latent TGFβ binding proteins (LTBPs), called a latent complex [23,24], which is crucial for the secretion of TGFβ and the activation of TGFβ receptor (TGFR)-mediated cell signaling [25,26]. TGFβ induces the expression of many other growth factors and cytokines to initiate EMT, while also cooperating with the initial stimulus of TGFβ to stimulate self-expression, which is necessary for sustained signaling, which continually supports the long process of EMT [22,27].

In this study, we investigate the potential effects of m^6^A on the TGFβ1-induced EMT of cancer cells. Our data reveal that TGFβ1-induced EMT is suppressed in METTL3 knockdown cells (Mettl3^Mut/−^). However, the expression of TGFβ1 is enhanced in Mettl3^Mut/−^ cells but decreased in TGFβ1-treated Mettl3^Mut/−^ cells. We demonstrate that m^6^A regulates the stability and translation of TGFB1 mRNA. In addition, METTL3 modulates the secretion and activation of TGFβ1. Besides this, we further reveal that the suppression of TGFβ1-induced EMT in Mettl3^Mut/−^ cells is mainly due to the downregulation of Snail. Together, our data suggest that m^6^A performs multi-functional roles in the TGFβ1 expression and EMT modulation of cancer cells.

## 2. Materials and Methods

### 2.1. Ethics Statement

All animal experiments were conducted according to the guidelines of the Animal Care and Use Committee of Sun Yat-sen University. Animals were maintained in accordance to the guidelines of the American Association of Laboratory Animal Care.

### 2.2. Cell Culture, Treatments, and Transfection

Mettl3^Mut/−^ cells were generated as previously described using the CRISPR-Cas9 editing system [28]. Both HeLa cells and Mettl3^Mut/−^ cells were cultured in Dulbecco’s Modified Eagle Medium (DMEM, GIBCO, Carlsbad, CA, USA) supplemented with 10% fetal bovine serum (FBS) at 37 °C in a 5% CO_2_ atmosphere. For TGFβ1 treatments, 10 ng/mL TGFβ1 (PeproTech, Rocky Hill, NJ, USA) was added into cell culture medium for the indicated time points. For transfection, plasmids were transfected into cells using Lipofectamine 3000 reagent (Invitrogen Life Technology, Carlsbad, CA, USA), following the manufacturer’s instructions.

### 2.3. Plasmid Constructions

The 5′UTR region of TGFB1 was cloned into the pGL3–basic vector in the front of the F-Luc gene (pGL3–basic–WT–5′UTR). The CDS region of TGFB1 was cloned into the pcDNA3–HisMax vector (pcDNA3–WT–CDS). Mutagenesis of m^6^A motifs (GGAC to GGCC) in both pGL3–basic–WT–5′UTR and pcDNA3–WT–CDS was performed using a site-directed mutagenesis kit (Thermo Fisher Scientific, Waltham, MA, US). Primers for mutagenesis are listed in Additional file 1: Table S1. The CDS region of the TGFβ binding protein (LTBP1) was cloned into the pHM6 vector.

### 2.4. Wound Healing Assay

Cells were seeded and cultured until 90% confluent monolayer. Cells were then scratched by a sterile pipette tip and applied for treatments in FBS-free medium, as indicated in the text. The migration distances of cells into the scratched area were measured in 10 randomly chosen fields under microscope.

### 2.5. In Vitro Invasion Assay

The transwell assay was conducted by CytoSelect™ 24-well Cell Invasion assay kits. Briefly, polycarbonate filters (8 µm pore size, Corning) coated with 50% Matrigel (BD bioscience, Bedford, MA) were used to separate the upper and lower chambers. Then, 5 × 10^4^ cells in 200 µL culture medium supplemented with 0.1% FBS were added into the upper chamber, while 600 µL medium supplemented with 10% FBS was added into the lower chamber and served as a chemotactic agent. For TGFβ1 treatments, 10 ng/mL TGFβ1 was supplemented in culture medium. After 48 h incubation, the invaded cells onto the lower chamber were fixed, stained, and counted under the upright microscope (five fields per chamber).

### 2.6. Western Blot Analysis

Total cell lysates were collected as previously described [29]. The primary antibodies used for immunoblotting included anti-TGFβ1 (21898-1-AP, Proteintech, Wuhan, China), anti-E-cad (ab1416, Abcam, Cambridge, MA, USA), anti-FN (sc-52331, Santa Cruz Biotechnology, Santa Cruz, CA, USA), anti-Snail (ab53519, Abcam), anti-METTL3 (ab195352, Abcam), anti-LTBP1 (A15287, Abclonal), anti-LTBP2 (PA5-51930, Invitrogen), anti-LTBP3 (A15687, Abclonal, Wuhan, China), anti-SMAD2 (ab40855, Abcam), anti-pSMAD2 (3108, Cell signaling technology, Danvers, MA, USA). The immunoblotting results presented were representatives from at least three independent experiments.

### 2.7. RNA Extraction and Quantitative Real-Time PCR (qRT-PCR)

RNA extraction with Trizol (Invitrogen) and real time PCR were performed according to the protocol used in our previous study [21]. The primers of targeted genes were as follow: TGFB1, forward 5′-CGCGTGCTAATGGTGGAAA-3′ and reverse 5′-CGCTTCTCGGAGCTCTGATG-3′; pre-TGFB1 forward 5′-CGTGCTAATGGTGGAAACCCA-3′ and reverse 5′-CAGTGCCATCCTCTTTCGGA-3′; HPRT, forward 5′-TGACACTGGCAAAACAATGCA-3′ and reverse 5′-GGTCCTTTTCACCAGCAAGCT-3′; GAPDH, forward 5′-GTCTCCTCTGACTTCAACAGC G-3′ and reverse 5′-ACCACCCTGTTGCTGTAGCCAA-3′; CDH1, forward 5′- GCCTCCTGAAAAGAGAGTGGAAG-3′ and reverse 5′- TGGCAGTGTCTCTCCAAATCC G-3′; 18S, forward 5′- CGGACAGGATTGACAGATTGATAGC-3′ and reverse 5′- TGCCAGAGTCTCGTTCGTTATCG-3′; MALAT1, forward 5′- GCTTGGCTTCTTCTGGACTCA-3′ and reverse 5′- TCGCGAGCTTCACCATGA-3′; 7SL, forward 5′- GCACTAAGTTCGGCATCAATATG-3′ and reverse 5′- AGTGCAGTGGCTATTCACAG-3′; VIM, forward 5′-AGGCAAAGCAGGAGTCCACTGA-3′ and reverse 5′-ATCTGGCGTTCCAGGGACTCAT-3′; SNAI1, forward 5′- TGCCCTCAAGATGCACATCCGA-3′ and reverse 5′- GGGACAGGAGAAGGGCTTCTC-3′; METTL3, forward 5′-CTATCTCCTGGCACTCGCAAGA-3′ and reverse 5′-GCTTGAACCGTGCAACCACATC-3′; LTBP1, forward 5′-GCCCTAATGGTGAGTGTTTGA-3′ and reverse 5′-AGATCACAGGGGGATCAGG-3′; LTBP2, forward 5′-TGCCCTAGTGGAAAAGGCTA-3′ and reverse 5′-TCACACACTCATCCGCATCT-3′; LTBP3, forward 5′-CACCTGAGGACACAGAGGAAG-3′ and reverse 5′-GAGATCAGCTCGGGGTAGG-3′; FLUC, forward 5′- GGCCTGACAGAAACAACCAG -3′ and reverse 5′- AAGTCCACCACCTTAGCCTC -3′; RLUC, forward 5′- CGCTATTGTCGAGGGAGCTA -3′ and reverse 5′- GCTCCACGAAGCTCTTGATG -3′; METTL3 (mouse), forward 5′- CAGTGCTACAGGATGACGGCTT-3′ and reverse 5′- CCGTCCTAATGATGCGCTGCAG-3′; GAPDH (mouse), forward 5′- CATCACTGCCACCCAGAAGACTG-3′ and reverse 5′- ATGCCAGTGAGCTTCCCGTTCAG-3′; TGFB1 (mouse), forward 5′- TGATACGCCTGAGTGGCTGTCT-3′ and reverse 5′- CACAAGAGCAGTGAGCGCTGAA-3′. GAPDH was used as a control for normalization.

### 2.8. m^6^A RNA-Immunoprecipitation (RIP) qPCR

The m^6^A-qRT-PCR was conducted according to previous studies, with slight modifications [30]. Briefly, total RNAs (200 ug) extracted by Trizol were used for immunoprecipitation by the m^6^A antibody (Synaptic Systems) or IgG in IP buffer (150 mM NaCl, 0.1% NP-40, 10 mM Tris, pH 7.4, 100U RNase inhibitor) to obtain the m^6^A pull down portion. The m^6^A RNAs were immunoprecipitated by Dynabeads^®^ Protein A (ThermoFisher Scientific) and eluted twice by elution buffer (5 mM Tris-HCL pH 7.5, 1 mM EDTA pH 8.0, 0.05% SDS, 20 mg/mL Proteinase K). m^6^A-IP RNAs were precipitated by ethanol and the RNA concentration was measured with a Qubit^®^ RNA HS Assay Kit (ThermoFisher Scientific). A total of 2 ng of total RNA (Input) and m^6^A-IP RNA were used as the template for qRT-PCR. HPRT1 acted as the internal control of the input samples, since it did not contain m^6^A peaks from the m^6^A profiling data [12]. The student’s *t*-test was used for statistical analysis.

### 2.9. Cell Fractionation Assay

A total of 10^7^ cells were rinsed with PBS once and then pelleted. Fractionation of the nuclear and cytoplasmic samples was performed using an NE-PER(R) nuclear and cytoplasmic extraction kit (Thermo Fisher). Total RNA in the nuclear and cytoplasmic fractions was extracted by Trizol. The nuclei–cytoplasm ratio was determined by the mRNA levels of targets in the nuclear and cytoplasmic fractions, which were normalized to the levels of nuclear MALAT1 RNA and cytoplasmic 7SL RNA, respectively [31].

### 2.10. mRNA Stability Assay

HeLa or Mettl3^Mut/−^ cells were seeded on six-well plate one day before treatment. A total of 5 µg/mL actinomycin D (Act-D) was added in serum-free culture medium for the indicated times. Cells were washed by PBS and subjected to isolate total RNA by Trizol. RNA concentrations were quantified by a Qubit^®^ RNA HS Assay Kit (ThermoFisher Scientific) and qRT-PCR performed, where 18S mRNA acted as the internal control.

### 2.11. Polysome Profiling

Polysome profiling was conducted according to previous studies [11]. Two 10 cm dishes of HeLa or Mettl3^Mut/−^ cells were incubated with 100 µg/mL cycloheximide (CHX) for 5 min at 37 °C before collection. The culture medium was removed and cells were washed with cold PBS with 100 µg/mL CHX. A total of 300 µL lysis buffer per dish (10 mM Tris, pH 7.4, 150 mM KCl, 5 mM MgCl_2_, 100 µg/mL CHX, 0.5% Triton X-100, freshly added protease inhibitor, and RNase inhibitor) was added to cells and kept on ice for 15 min with pipetting. Cell suspension was centrifuged at 13,000g for 15 min, and supernatant was collected and tested at 260 nm. A 5/50% *w*/*v* sucrose gradient was prepared in lysis buffer without Triton X-100. Clear cell lysate was loaded on the sucrose gradient and centrifuged at 4 °C for 4 h at 27,500 rpm. The sample was then fractioned and analyzed by Gradient Station (BioCamp, New Brunswick, Canada). Fractionated samples were then used to isolate total RNA for qRT-PCR.

### 2.12. Dual-Luciferase Reporter Assay

The luciferase assay was performed using reporter lysis buffer (Promega, Milan, Italy) and luciferase assay reagents according to the manufacturer’s instructions. Briefly, cells were co-transfected with pGL3–basic–WT–5′UTR or pGL3–basic–Mut–5′UTR and TK-Rluc reporter in six-well plate for 24 h. Cells were then analyzed with the Dual-Glo Luciferase Assay system (Promega). Renilla Luciferase (R-Luc) was used to normalize firefly luciferase (F-Luc) activity.

### 2.13. TGFβ1 Secretion Detection

Cell culture medium was collected after 48 h incubation with TGFβ1. TGFβ1 secretion was detected by a TGFβ1 ELISA kit, following the manufacturer’s instruction (Quantikine ELISA Kits, R & D systems, Minneapolis, MN, USA). The results were calculated by reference to the standard curve provided by the manufacturer standards, within the range from 0 pg/mL to 2000 pg/mL. Secreted TGFβ1 levels from cells with or without treatment were normalized to plain culture medium or TGFβ1-treated culture medium, respectively.

## 3. Results

### 3.1. METTL3 Is Essential for TGFβ1-Induced EMT

**Figure 1:**
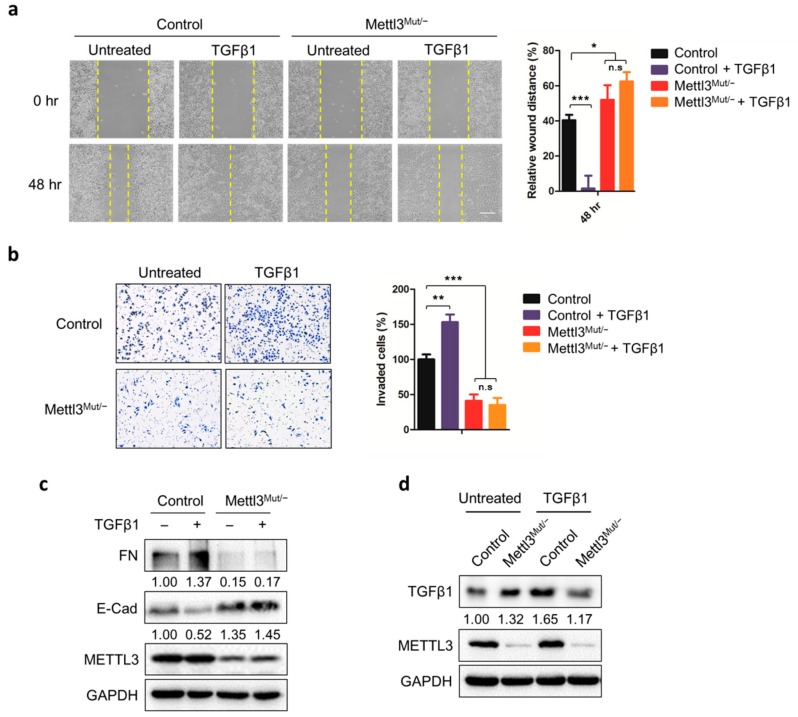
METTL3 regulates EMT and TGFβ1 expression in HeLa cells. (**a**) Control and Mettl3^Mut/−^ HeLa cells were incubated with 10 ng/mL TGFβ1 for indicated times. The wound healing of cells was recorded (left) and quantitatively analyzed (right); scale bar, 100 µm; (**b**) Control and Mettl3^Mut/−^ HeLa cells were incubated with 10 ng/mL TGFβ1 and cells were allowed to invade for 24 h. Invaded cells were tested by CytoSelect™ 24-well Cell Invasion assay kits (8 µm, colorimetric format; left) and quantitatively analyzed (right); (**c**) Control and Mettl3^Mut/−^ HeLa cells were incubated with 10 ng/mL TGFβ1 for 48 h. Protein levels of fibronectin (FN) and E-cadherin (E-Cad) were measured by Western blot. The band intensities of FN and E-Cad were analyzed by ImageJ and listed at the bottom of target bands; (**d**) Control and Mettl3^Mut/−^ HeLa cells were incubated with 10 ng/mL TGFβ1 for 48 h. The protein levels of TGFβ1 in control and Mettl3^Mut/−^ HeLa cells were measured by Western blot. Band intensities of TGFβ1 were analyzed by ImageJ and listed at the bottom of TGFβ1 bands. Data are presented as means ± SD from three independent experiments. Student’s *t*-test, n.s, no significant; *, *p* < 0.05; **, *p* < 0.01; ***, *p* < 0.001 compared with control. +, with treatment; −, without treatment.

To verify the effect of METTL3 in HeLa cells, we constructed METTL3 knockdown HeLa cells (Mettl3^Mut/−^) by CRISPR/Cas9 system for studies. The enzymatic activities of METTL3 were detected by LC-MS/MS (Additional file 1: Figure S1a) [28]. Results showed that both the cell migration and cell invasion abilities of Mettl3^Mut/−^ cells decreased significantly compared to control cells (Figure 1a,b). Consistently, the down-regulation of fibronectin (FN) and up-regulation of E-cadherin (E-cad) were observed in Mettl3^Mut/−^ cells (Figure 1c), indicating that METTL3 modulated both cell migration and invasion in HeLa cells.

To further investigate the effect of METTL3 on EMT, we treated cells with 10 ng/mL TGFβ1, which has been considered to be the major EMT inducer in cancer cells. Both wound healing assay and Transwell assay showed that cell migration and invasion of control HeLa cells were successfully enhanced by TGFβ1, while there was no significant difference in Mettl3^Mut/−^ cells (Figure 1a,b). Western blot analysis showed up-regulation of FN and down-regulation of E-cad in TGFβ1-treated HeLa cells; however, neither FN or E-cad in TGFβ1-treated Mettl3^Mut/−^ cells showed significant change (Figure 1c), suggesting that the EMT process in Mettl3^Mut/−^ cells was inhibited even in the presence of TGFβ1. We further re-introduced METTL3 into Mettl3^Mut/−^ cells, TGFβ1-treated Mettl3^Mut/−^ cells regained both cell migration and invasion abilities (Additional file 1: Figure S1b,c). Nevertheless, up-regulation of FN and down-regulation of E-cad were observed in Mettl3^Mut/−^ cells overexpressing METTL3, and TGFβ1 treatments further enhanced these trends (Additional file 1: Figure S1d). Together, our data suggest that METTL3 was essential for TGFβ1-induced EMT.

By comparing TGFβ1 expressions between control and Mettl3^Mut/−^ cells, Western blot results surprisingly showed that the expression of TGFβ1 increased in Mettl3^Mut/−^ cells (Figure 1d). Furthermore, up-regulation of TGFβ1 upon extracellular TGFβ1 stimulation was observed in control HeLa cells, which is in line with previous reports (Figure 1d) [32]. However, up-regulation of TGFβ1 was no longer observed in Mettl3^Mut/−^ cells after TGFβ1 stimulation, indicating that METTL3 was involved in the expression of TGFβ1.

### 3.2. m^6^A Negatively Regulates the mRNA Stability of TGFB1

**Figure 2:**
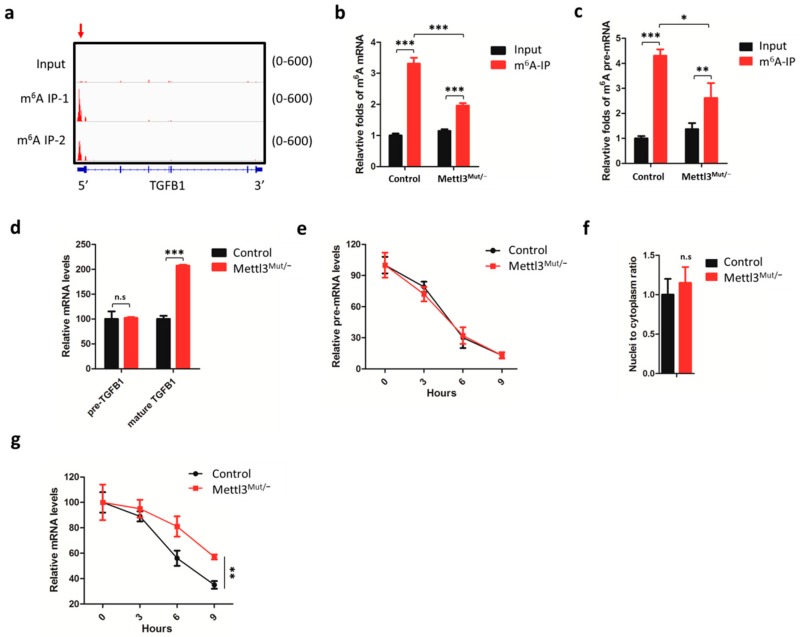
m^6^A triggers TGFB1 mRNA decay in HeLa cells. (**a**) m^6^A peaks were enriched in the 5′UTR and CDS regions of TGFB1 mRNA from m^6^A RIP-seq data. Arrows marked the m^6^A peaks in HeLa cells. The m^6^A abundance of TGFB1 mRNA is listed on the right; (**b**) m^6^A RIP-qPCR analysis of TGFB1 mature mRNA in control and Mettl3^Mut/−^ HeLa cells. Input samples were total RNA extracted from control and Mettl3^Mut/−^ HeLa cells. Relative folds of TGFB1 mRNA in m^6^A RIP samples were normalized to the input of control cells; (**c**) m^6^A RIP-qPCR analysis of TGFB1 precursor (pre-) mRNA in control and Mettl3^Mut/−^ HeLa cells. Relative folds of TGFB1 pre-mRNA in m^6^A RIP samples were normalized to the input of control cells; (**d**) expression levels of precursor (pre-) and mature mRNA of TGFB1 in control and Mettl3^Mut/−^ HeLa cells were measured by qRT-PCR; (**e**) Control and Mettl3^Mut/−^ HeLa cells were incubated with 5 µg/mL Act-D for indicated times. Expression levels of TGFB1 pre-mRNA were measured by qRT-PCR; (**f**) TGFB1 mRNA levels in nuclear and cytoplasmic fractions from control and Mettl3^Mut/−^ HeLa cells were measured by qRT-PCR. Localization of TGFB1 mRNA was calculated as nuclear abundance divided to cytoplasmic abundance; (**g**) Control and Mettl3^Mut/−^ HeLa cells were incubated with 5 µg/mL Act-D for indicated times. Expression levels of TGFB1 mRNA were measured by qRT-PCR. Data are presented as means ± SD from three independent experiments. Student’s *t*-test, n.s, no significant; *, *p* < 0.05; **, *p* < 0.01; ***, *p* < 0.001 compared with control.

Considering that METTL3 is critical to mRNA m^6^A methylation, we questioned whether the m^6^A of TGFβ1 mRNA regulated its expression. Firstly, we assumed from the m^6^A sequencing results from our previous study (Accession code GSE112795) [21] that TGFB1 mRNA contained two m^6^A modification peaks in 5′UTR and exon 1, respectively (Figure 2a). We performed m^6^A RNA-immunoprecipitation (RIP) qPCR and confirmed that m^6^A levels of TGFB1 mRNA reduced significantly in Mettl3^Mut/−^ cells (Figure 2b). Similarly, the m^6^A levels of TGFB1 precursor (pre-) mRNA decreased in Mettl3^Mut/−^ cells (Figure 2c). Our data suggested that METTL3 was responsible for the m^6^A modifications on both TGFB1 mature mRNA and pre-mRNA. By examining the expression of TGFB1 mRNA, qRT-PCR results showed that mature TGFB1 levels enhanced significantly (Figure 2d), indicating that m^6^A methylation regulated TGFβ1 expression.

Furthermore, we observed that there was no significant differences in pre-mRNA between control and Mettl3^Mut/−^ cells (Figure 2d), suggesting that the pre-mRNA levels of TGFB1 were not affected by m^6^A methylation. Next, we compared the clearance and splicing rate of precursor mRNA (Figure 2e), nuclei-cytoplasm transport (Figure 2f), and stability of TGFB1 mRNA (Figure 2g) between control and Mettl3^Mut/−^ cells. Results showed that the knockdown of METTL3 had no effect on the clearance and splicing rate of precursor mRNA or nuclei-cytoplasm transport of TGFB1 mRNA (Figure 2e,f; Additional file 1: Figure S2). However, the half-life of TGFB1 mature mRNA was longer in Mettl3^Mut/−^ cells compared to control cells, suggesting that m^6^A methylation negatively regulated TGFB1 mRNA decay (Figure 2g).

### 3.3. m^6^A Negatively Regulates the Translation Elongation of TGFB1 mRNA

**Figure 3:**
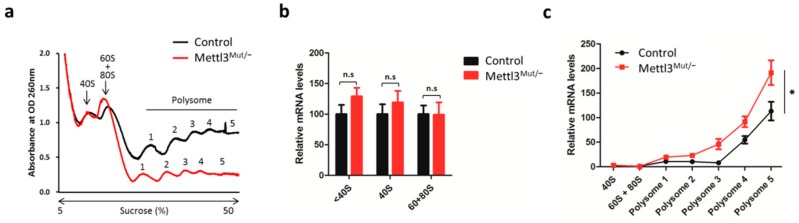
m^6^A modulates translation elongation of TGFB1 mRNA in HeLa cells. (**a**) Polysome profiles of control and Mettl3^Mut/−^ HeLa cells. Numbers in polysome fraction represent the samples that were used for qRT-PCR in Figure 3c; (**b**) Expression levels of TGFB1 mRNA in ribosome-unbound (<40S), 40S, and 60 + 80S fractions from control and Mettl3^Mut/−^ HeLa cells; (**c**) Expression levels of TGFB1 mRNA in 40S, 60 + 80S and polysome fractions from control and Mettl3^Mut/−^ HeLa cells. Data are presented as means ± SD from three independent experiments. Student’s *t*-test, n.s, no significant; *, *p* < 0.05 compared with control.

We further investigated whether METTL3 may regulate the translation progression of TGFβ1. The effect of METTL3 on the translation process of TGFB1 mRNA was investigated by separating RNA fractions via polysome profiling from control and Mettl3^Mut/−^ cells (Figure 3a). RNA fractionation results suggest that m^6^A modification did not affect the translation initiation process (40S, 60S, and 80S) of TGFB1 mRNA (Figure 3b), but severally enhanced the elongation process (polysome fractions, Figure 3c) in Mettl3^Mut/−^ cells. It indicated that m^6^A may negatively regulate the translation elongation of TGFB1 mRNA.

### 3.4. m^6^A in 5′UTR and CDS Mediates METTL3-Suppressed Translation of TGFB1

**Figure 4:**
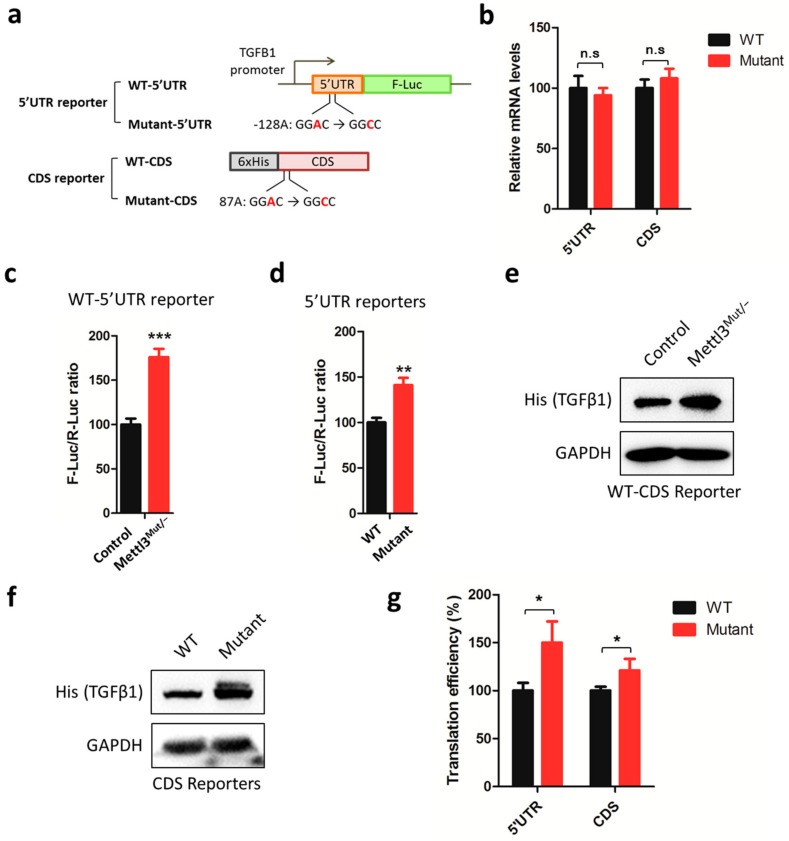
m^6^A methylation on both the 5′UTR and CDS regions of TGFB1 mRNA controls its translation efficiency. (**a**) Reporters for 5′UTR and CDS regions of TGFB1 mRNA. Potential m^6^A sites were mutated (GGAC to GGCC); (**b**) wild type (WT) and mutated reporters were transfected in HeLa cells for 48 h. Expression levels of reporter mRNA were measured by qRT-PCR: FLUC mRNA for 5′UTR reporter, normalized to RLUC mRNA levels; TGFB1 mRNA for the CDS reporter, normalized to GAPDH mRNA levels; (**c**) the WT-5′UTR reporter was co-transfected with TK-Rluc reporter in control and Mettl3^Mut/−^ HeLa cells for 48 h. Dual-luciferase assay was performed to measure F-Luc production, which was normalized to R-Luc levels; (**d**) WT-5′UTR or Mutant-5′UTR reporter were co-transfected with TK-Rluc reporter in HeLa cells for 48 h. Dual-luciferase assay was performed to measure F-Luc production, which was normalized to R-Luc levels; (**e**) WT-CDS reporter was transfected in control and Mettl3^Mut/−^ HeLa cells for 48 h. Expression levels of exogenous TGFβ1 (His) were measured by Western blot; (**f**) WT-CDS or Mutant-CDS reporter were transfected in HeLa cells for 48 h. Expression levels of exogenous TGFβ1 (His) were measured by Western blot; (**g**) Translation efficiency of WT and TGFβ1 mutant is defined as the quotient of reporter protein production divided by mRNA abundance [11]. For 5′UTR, reporter protein production was determined from dual-luciferase assay; for CDS, reporter protein production was analyzed by ImageJ from Western blot. Data are presented as means ± SD from three independent experiments. Student’s *t*-test, n.s, no significant; *, *p* < 0.05; **, *p* < 0.01; ***, *p* < 0.001 compared with control.

We further investigated the potential methylation sites responsible for m^6^A regulated expression of TGFβ1. Firstly, m^6^A-seq data revealed that there was significant enrichment of m^6^A in its CDS and 5′UTR regions (Figure 2a). Thus, two reporters that contained the TGFB1 5′UTR and CDS regions were generated: 1) the 5′UTR region was constructed into the pGL3–basic vector before F-Luc gene, while 2) the CDS region was constructed into the mammalian expression vector. Combining the gene information (Additional file 1: Figure S3a) and m^6^A distribution (Figure 2a) of TGFB1 mRNA, two potential m^6^A sites were targeted: –128A in 5′UTR and 87A in CDS were mutated for study (Figure 4a). To minimize different transfection efficiencies of reporters between control and Mettl3^Mut/−^ cells, transfection amounts of reporters were adjusted. Besides this, mRNA levels of wild-type and mutated reporters were detected in HeLa cells, but no significant difference was observed in both 5′UTR and CDS reporters (Figure 4b).

Firstly, dual-luciferase assay showed that the luciferase production of wild-type 5′UTR reporter was increased in Mettl3^Mut/−^ cells (Figure 4c). Consistently, 5′UTR reporters containing m^6^A mutation also showed increased relative F-Luc values compared to that of wild type reporters (Figure 4d), while there was no significant difference between wild type and mutant reporters in Mettl3^Mut/−^ cells (Additional file 1: Figure S3b). Secondly, Western blot analysis confirmed that the TGFB1 wild-type CDS was increased in Mettl3^Mut/−^ cells (Figure 4e) and the m^6^A mutation can increase the expression of TGFB1 (Figure 4f, Additional file 1: Figure S3c). We further analyzed the translation efficiency by calculating the quotient of reporter protein production divided by mRNA abundance [11]. The results showed that mutations in both 5′UTR and CDS could enhance the translation efficiency of targets (Figure 4g). All these data suggest that m^6^A on TGFB1 mRNA 5′UTR and CDS can suppress its translation, while it has no effect on its mRNA expression.

### 3.5. Protein Stability, Secretion, and Activation of TGFβ1 Decreased in Mettl3^Mut/−^ Cells

**Figure 5:**
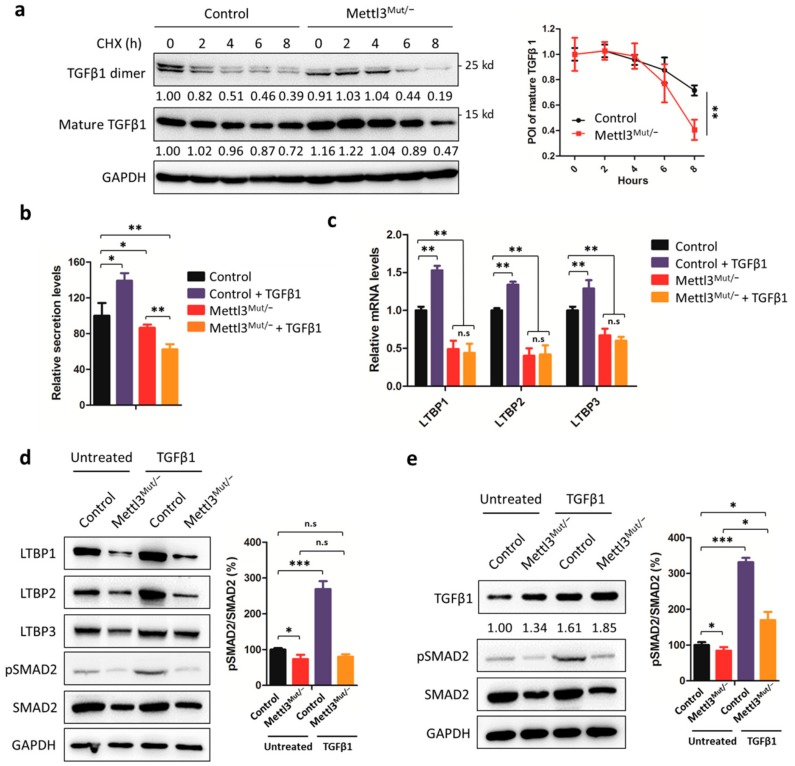
Secretion of TGFβ1 is modulated by METTL3. (**a**) Control and Mettl3^Mut/−^ HeLa cells were incubated with 100 µg/mL cycloheximide (CHX) for indicated times. Protein levels of mature TGFβ1 and TGFβ1 dimer were measured by Western blot (left). Band intensities were analyzed by ImageJ and are listed at the bottom of target bands. Mature TGFβ1 levels were quantitatively analyzed (right); (**b**) Control and Mettl3^Mut/−^ HeLa cells were incubated with 10 ng/mL TGFβ1 for 48 h. Secretion of TGFβ1 in control and Mettl3^Mut/−^ HeLa cells was measured by ELISA kit. The relative secretion levels of TGFβ1 were normalized to culture medium with or without the addition of TGFβ1; (**c**) Control and Mettl3^Mut/−^ HeLa cells were incubated with 10 ng/mL TGFβ1 for 48 h. The expression levels of LTBP1, LTBP2, and LTBP3 mRNA in control and Mettl3^Mut/−^ HeLa cells were measured by qRT-PCR; (**d**) Control and Mettl3^Mut/−^ HeLa cells were incubated with 10 ng/mL TGFβ1 for 48 h. The expression levels of LTBP1, LTBP2, LTBP3, pSMAD2, and SMAD2 in control and Mettl3^Mut/−^ HeLa cells were measured by Western blot (left). Percentages of pSMAD2 to SMAD2 were analyzed (right); (**e**) Control and Mettl3^Mut/−^ HeLa cells were transiently overexpressed in LTBP1 for 24 h, then incubated with 10 ng/mL TGFβ1 for 48 h. The expression levels of TGFβ1, pSMAD2, and SMAD2 were measured by Western blot (left). Band intensities of TGFβ1 were analyzed by ImageJ and are listed at the bottom of targets. Percentages of pSMAD2 to SMAD2 were analyzed (right). Data are presented as means ± SD from three independent experiments. Student’s *t*-test, n.s, no significant; *, *p* < 0.05; **, *p* < 0.01; ***, *p* < 0.001 compared with control.

Next, we examined the protein stability of TGFβ1 by incubating cells with protein synthesis inhibitor cycloheximide (CHX). The Western blot results indicate that either the TGFβ1 monomer (mature TGFβ1) or dimer showed a shorter protein half-life in Mettl3^Mut/−^ cells (Figure 5a). Similar results were observed in cells treated with TGFβ1 (Additional file 1: Figure S4a). Notably, we observed a 78.4 ± 3.2% reduction of TGFβ1 dimer formation in Mettl3^Mut/−^ cells, compared to TGFβ1 monomer protein amount (Figure 5a), hinting that the secretion of TGFβ1 might be regulated by METTL3. To verify our hypothesis, we detected extracellular TGFβ1 levels by ELISA kit. The results showed that there was 86.5 ± 3.5% less TGFβ1 secretion in Mettl3^Mut/−^ cells compared to control (Figure 5b). After treatment of TGFβ1, the inducing effect of TGFβ1 secretion was no longer observed in Mettl3^Mut/−^ cells; rather, it showed a 62.3 ± 5.8% drop compared to untreated Mettl3^Mut/−^ cells.

Considering that the TGFβ1 dimer formation and secretion are regulated by latent TGFβ binding proteins (LTBPs), we then compared the expression levels of LTBP1, LTBP2, and LTBP3 between control and Mettl3^Mut/−^ cells. qRT-PCR and Western blot results consistently showed that all tested LTBPs decreased significantly in Mettl3^Mut/−^ cells (Figure 5c,d). Furthermore, TGFβ1-induced up-regulation of LTBP levels in control cells was no longer observed in Mettl3^Mut/−^ cells (Figure 5c,d), suggesting that METTL3 regulated the expression of LTBPs. To confirm this suggestion, we transiently overexpressed LTBP1, which is the most well studied LTBP, in control and Mettl3^Mut/−^ cells (Additional file 1: Figure S4b). Western blot results (Figure 5e) showed that the TGFβ1 expression patterns were similar to the untransfected cells shown in Figure 1d. Upon TGFβ1 stimulation, LTBP1 up-regulated TGFβ1 levels in Mettl3^Mut/−^ cells as well as control, suggesting that the expression of TGFβ1 was recovered (Figure 5e).

Since the secretion of TGFβ1 is necessary to the activation of TGFβ1/Smad pathway, we detected phosphorylation of SMAD2 (pSMAD2) in both control and Mettl3^Mut/−^ cells to investigate whether TGFβ1 activation was regulated by METTL3. Interestingly, both total SMAD2 levels and the pSMAD2 to SMAD2 ratio decreased in Mettl3^Mut/−^ cells (Figure 5d). Upon TGFβ1 treatments, pSMAD2 to SMAD2 ratio in Mettl3^Mut/−^ cells failed to increase as those in control cells (Figure 5d), suggesting that the activation of TGFβ1 was inhibited in cells lacking METTL3. After re-introducing LTBP1, the pSMAD2 to SMAD2 ratio in Mettl3^Mut/−^ cells partially increased from 84.57 ± 10.02% to 169.91 ± 23.11% upon TGFβ1 treatments (Figure 5e), suggesting that LTBP1 was not the unique factor affecting TGFβ1 activation. Taken together, our results indicate that protein half-life, secretion, and activation of TGFβ1 were suppressed in Mettl3^Mut/−^ cells.

### 3.6. Snail Is the Key Factor Responsible for TGFβ1-Induced EMT in Mettl3^Mut/−^ Cells

**Figure 6:**
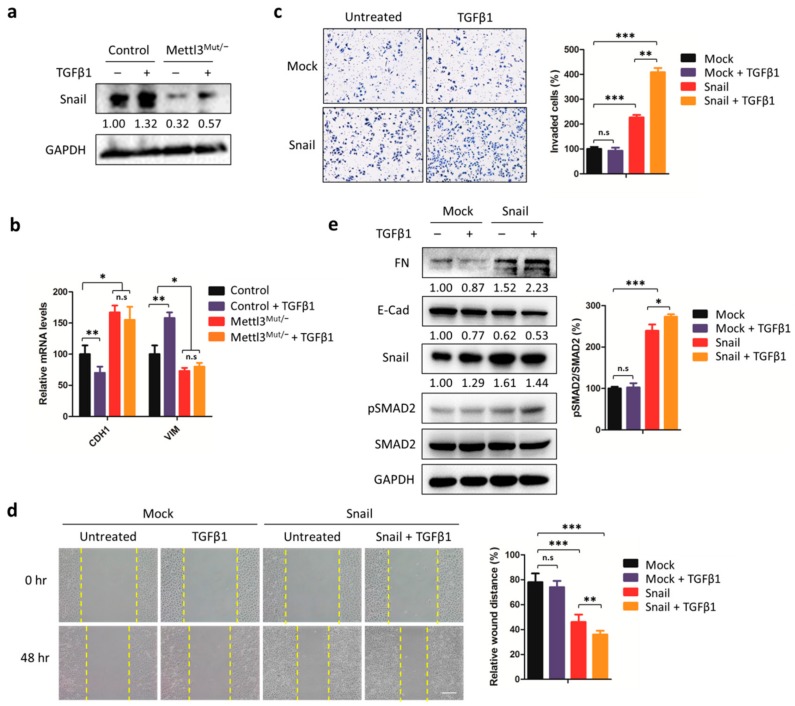
Snail is the core protein responsible for METTL3-mediated EMT. (**a**) Control and Mettl3^Mut/−^ HeLa cells were incubated with 10 ng/mL TGFβ1 for 48 h. The expression levels of Snail were measured by Western blot. Band intensities of Snail were analyzed by ImageJ and are listed at the bottom of target bands; (**b**) Control and Mettl3^Mut/−^ HeLa cells were incubated with 10 ng/mL TGFβ1 for 48 h. Expression levels of CDH1 and VIM mRNA were measured by qRT-PCR; (**c**) Mettl3^Mut/−^ cells transiently overexpressing empty vector (Mock) and Snail were incubated with 10 ng/mL TGFβ1. Cells were allowed to invade for 24 h and were tested by CytoSelect™ 24-well Cell Invasion assay kits (8 µm, colorimetric format; left). Invaded cells were then quantitatively analyzed (right); (**d**) Mettl3^Mut/−^ cells transiently overexpressing empty vector (Mock) and Snail were incubated with 10 ng/mL TGFβ1 for indicated times, and the wound healing of cells was recorded; scale bar, 100 µm; (**e**) Mettl3^Mut/−^ cells transiently overexpressing empty vector (Mock) and Snail were incubated with 10 ng/mL TGFβ1 for 48 h. Expression levels of FN, E-Cad, Snail, pSMAD2, and SMAD2 were measured by Western blot (left). Band intensities of FN, E-Cad, and Snail were analyzed by ImageJ and listed at the bottom of targets. Percentages of pSMAD2 to SMAD2 were analyzed (right). Data are presented as means ± SD from three independent experiments. Student’s *t*-test, n.s, no significant; *, *p* < 0.05; **, *p* < 0.01; ***, *p* < 0.001 compared with control.

Furthermore, we questioned why TGFβ1 did not stimulate EMT in Mettl3^Mut/−^ cells. Since the TGFβ1-stimulated EMT process can be mediated by Smad signaling, which activates the transcription of EMT transcription factor (EMT-TF) Snail, we verified the expressions of Snail in both control and Mettl3^Mut/−^ cells. As expected, Snail protein levels increased in control cells treated with TGFβ1 (Figure 6a). However, a significant down-regulation of Snail protein was observed in Mettl3^Mut/−^ cells, and TGFβ1 stimulation could only increase the limited amount of Snail proteins (Figure 6a). Consistently, the expression of two EMT markers, CDH1 and VIM, which were direct downstream genes of Snail [33], was abolished in TGFβ1-treated Mettl3^Mut/−^ cells (Figure 6b). Our results suggest that the inhibition of TGFβ1-induced EMT in Mettl3^Mut/−^ cells may due to the down-regulation of Snail.

In order to reverse the effect of Snail down-regulation in Mettl3^Mut/−^ cells, we transiently overexpressed Snail and further examined its effect on cell invasion and migration in vitro. Transwell assay showed that Mettl3^Mut/−^ cells overexpressing Snail showed stronger cell invasion ability (Figure 6c). After TGFβ1 stimulation, cell invasion was significantly enhanced in Mettl3^Mut/−^ cells overexpressing Snail, but not in Mettl3^Mut/−^ cells transfected with empty vector (Mock). Similar results were obtained in wound-healing assays, showing that Mettl3^Mut/−^ cells overexpressing Snail regained the cell migration ability and TGFβ1 treatment further stimulated the migration effect (Figure 6d). Western blot analysis consistently indicated that the overexpression of Snail in Mettl3^Mut/−^ cells induced an increase of FN and decrease of E-cad, and the trends of both FN and E-cad in TGFβ1-treated cells were more obvious than non-treated cells (Figure 6e). By detecting the SMAD2 phosphorylation, results showed that the overexpression of Snail recruited pSMAD2 levels, and TGFβ1 treatments further enhanced the pSMAD2 to SMAD2 ratio (Figure 6e), indicating the TGFβ1 signaling was activated after re-introducing Snail. Together, our results suggest that TGFβ1 can successfully induce the EMT process in Mettl3^Mut/−^ cells overexpressing Snail via Smad signaling.

### 3.7. METTL3/ TGFβ1/Snail Axis Regulates the In Vivo Progression of Cancer

**Figure 7:**
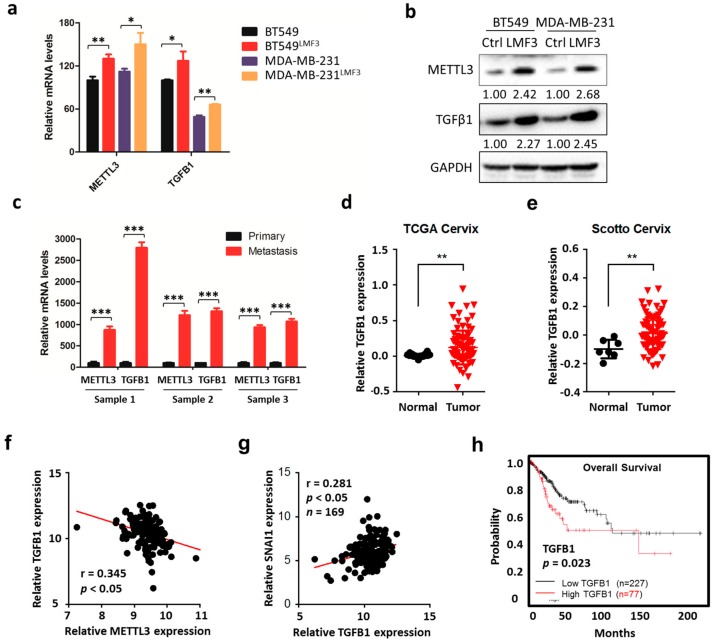
METTL3/ TGFβ1/Snail axis regulates the in vivo progression of cancer. (**a**) Expression levels of METTL3 and TGFB1 mRNA in MDA-MB-231^LMF3^ or BT-549 ^LMF3^ cells and their corresponding parental cells were measured by qRT-PCR; (**b**) expression levels of METTL3 and TGFβ1 in MDA-MB-231^LMF3^ or BT-549 ^LMF3^ cells (LMF3) and their corresponding parental cells (Ctrl) were measured by Western blot. Band intensities of METTL3 and TGFβ1 were analyzed by ImageJ and are listed at the bottom of target bands; (**c**) expression levels of METTL3 and TGFB1 mRNA in primary tumors and metastasized tumors in lungs isolated from three MMTV-PyMT mice were measured by qRT-PCR. Data are presented as means ± SD from three technical replicates. (**d**) Expression of TGFB1 in cervical tumor tissues and normal tissues from Oncomine database: Ma Breast 4; (**e**) Expression of TGFB1 in cervical tumor tissues and normal tissues from Oncomine database: Cluck Breast; (**f**) Pearson correlation between TGFB1 and METTL3 in 169 cervical cancer tissues from the TCGA database; (**g**) Pearson correlation between TGFB1 and SNAI1 in 169 cervical cancer tissues from the TCGA database; (**h**) Overall survival (OS) in patients with high (*n* = 227) versus low (*n* = 77) levels of TGFB1 in cervical cancer patients plotted by the Kaplan–Meier method. Data in Figure 7a are presented as means ± SD from three independent experiments. Student’s *t*-test, *, *p* < 0.05; **, *p* < 0.01 compared with control.

We further tested the potential effects of the METTL3/TGFβ1/Snail axis on the in vivo progression of cancer. We established high lung metastasis potential cancer cell models in our previous studies [21,34] and named them MDA-MB-231^LMF3^ or BT-549 ^LMF3^ cells. Both qRT-PCR and Western blot analysis showed that the expression of TGFβ1 and METTL3 increased in high lung metastasis potential cancer cells compared with that in the parental cells (Figure 7a,b). To confirm these observations, we used a mammary tumor virus (MMTV) promoter-driven polyoma middle T antigen (PyMT) mice model, which tends to develop secondary metastatic tumors in lungs in tumor-bearing mice [35]. qRT-PCR showed enhanced expression of METTL3 and TGFβ1 in metastasized lung tumors compared to that in the primary tumors isolated from MMTV-PyMT mice (Figure 7c), confirming that METTL3/TGFβ1 triggers cancer progression and metastasis.

We then questioned the possibility of a link between the METTL3/TGFβ1/Snail axis and the clinical development of cervical cancer. TGFB1 expression in cervical cancer tissues was significantly (*p* < 0.01) greater than that in normal tissues, according to TCGA Cervix (Figure 7d) and Scotto Cervix (Figure 7e) data from the Oncomine database. Furthermore, the mRNA expression of TGFB1 was negatively correlated with the expression of METTL3 (Figure 7f), while positively correlated with the expression of SNAI1 (Figure 7g) in 169 cases of cervical cancer patients. Using the online bioinformatics tool Kaplan–Meier plotter [36], we found that cervical cancer patients with increased expression of TGFB1 showed reduced overall survival (OS, Figure 7h). Together, these data suggested that METTL3/ TGFβ1/Snail axis regulates the in vivo progression of cancer.

## 4. Discussion

In the present study, we showed that TGFβ1 failed to induce EMT in Mettl3^Mut/−^ cells, and the TGFβ1-induced up-regulation of TGFβ1 was inhibited. On one hand, we found that the expression of TGFβ1 was modulated by m^6^A, and that m^6^A methylation on TGFB1 mRNA enhanced its mRNA decay and reduced translation. Besides this, the dimer formation of TGFβ1, which is necessary for its autocrine, was suppressed in Mettl3^Mut/−^ cells. On the other hand, we demonstrated that a lack of METTL3 suppressed activation of TGFβ1, and the down-regulation of Snail in Mettl3^Mut/−^cells was responsible for the failure of the TGFβ1-induced EMT process.

Increasing evidence suggests that METTL3 is involved in the progression of cancer cells [18,37,38]. Consistent with previous report [21], we revealed that TGFβ1 failed to induce EMT in the absence of METTL3, indicating the critical role of METTL3 in the EMT process of HeLa cells. Furthermore, SMAD2 and Snail, effectors of TGFβ1 signaling and EMT-inducing transcription factor, respectively, were down-regulated in Mettl3^Mut/−^ cells, indicating that the abolishment of TGFβ1 signaling and suppressed translation of Snail [21] are related to this EMT inhibition. TGFβ signaling plays a prominent role in EMT development, among a plethora of signaling pathways [22]. Here, we reported that METTL3 modulated TGFβ1 signaling both extracellularly and intracellularly. On one hand, down-regulation of LTBPs decreased free TGFβ1 binding to receptors, which therefore suppressed the TGFβ1 signaling initiation [39]. On the other hand, both SMAD2 and pSMAD2 were down-regulated in Mettl3^Mut/−^ cells, suggesting the TGFβ/Smad signal was inhibited. Despite our observations, a novel link between SMADs and m^6^A methylation has been reported recently, showing that SMAD2/3 colocalizes with the METTL3–METTL14–WTAP complex and promotes binding of the m^6^A methyltransferase complex onto targets [40]. This shows the possibility that there may be a mutual benefit between TGFβ/Smad signal and m^6^A methylation, and co-regulation of biological processes, including EMT.

m^6^A methylation-modulated EMT via Snail expression has been reported [21]. Here, we consistently showed that Mettl3^Mut/−^ cells transiently recruiting Snail performed TGFβ1-induced EMT behaviors, suggesting the critical role of Snail in TGFβ1-induced EMT. The TGFβ1-induced transition of EMT is accomplished by Smad signals, which enhances the Snail expression and further activates the transcription of EMT factors, such as down-regulation of E-cad and up-regulation of Vim [33]. Notably, we observed that the overexpression of Snail can enhance pSMAD2 levels, which was inhibited in Mettl3^Mut/−^ cells, indicating that there is a positive regulation of Snail on Smad signaling. Furthermore, we showed that there is strong correlation between the METTL3/TGFβ1/Snail axis and cancer progression in lung metastasis potential cancer cell models. By analyzing clinical data of cervical cancer, the correlation between METTL3 and TGFβ1 was relatively weak, which was mainly due to the inclusion of primary cervical cancer cases. Increasing reports show that metastatic dissemination can occur from the earliest point of tumorigenesis, prior to the clinical manifestation of tumors [41,42,43], indicating that the primary tumors also have metastatic potential. However, the TGFβ1 levels in primary cancer are commonly lower than in metastatic cancer [44], which therefore may affect the correlation analysis. Together, our data demonstrated that both METTL3 and Snail play key roles in responding to TGFβ1 signals in HeLa cells undergoing the EMT process.

Functions of m^6^A on mRNA translation have been studied for years. However, the exact role of m^6^A on the translation process is unknown. Wang et al. [11] revealed that 3′UTR m^6^A recruited reader protein YTHDF1 and promoted the circulation of mRNA to improve translation efficiency. Zhou et al. [45] demonstrated that 5′UTR m^6^A regulated the reinitiation and controlling alternative translation of ATF4 mRNA. Later, Lin et al. [21] revealed that CDS m^6^A triggered SNAI translation via YTHDF1 and eEF-2 interaction. It seems that localization of m^6^A may be critical to the translation regulation of their targets. Here, we reported that TGFB1 translation was inhibited when m^6^A modification existed. According to m^6^A-seq results (Figure 2a), two obvious m^6^A modification signals were observed in 5′UTR and at the beginning of the CDS of TGFB1 mRNA, with 386.3 ± 88.2% and 115.5 ± 33.5% abundance, respectively. Interestingly, mutations of potential m^6^A methylation sites in both 5′UTR and CDS can enhance target expressions (Figure 4d,f), which was due to the increase of translation efficiency (Figure 4g). It is possible that m^6^A methylation on TGFB1 mRNA decreases the elongation efficiency during the translation process (Figure 3c). However, the detailed mechanism of how m^6^A methylation regulation TGFβ1 translation remains to be elucidated.

TGFβ1 can autocrine from cells and further stimulates its own expression through the up-regulation of TGFβ receptors, which stimulate TGFβ1 expression and sustain signaling for the long process of EMT [22,27,32]. The activation of TGFβ is achieved by the cleavage of TGFβ propeptide, called the latency-associated protein (LAP), which is accomplished by LTBP interaction [46,47]. After dissociating TGFβ from LAP, TGFβ is secreted in a latent form, containing LTBP, TGFβ dimer, and non-covalent binding LAPs. LTBPs are essential to maintain TGFβ latency and target the latent TGFβ to extracellular matrix (ECM) [39]. Our data revealed that expressions of LTBPs were suppressed in Mettl3^Mut/−^ cells, even under TGFβ1 stimulation conditions, leading to failure of the TGFβ1 dimer formation, therefore inhibiting the TGFβ1 activation. This hypothesis was further confirmed by the rescue experiment, which shows that the overexpression of LTBP1 can recover the TGFβ1 expression in Mettl3^Mut/−^ cells (Figure 5e). Based on our m^6^A-seq results (accession code GSE112795) [21], only LTBP3 contained significant m^6^A modifications on its mRNA (Additional file 1: Figure S5), indicating that METTL3 probably regulated LTBP expression indirectly, despite modulating mRNA behaviors of LTBPs. Remarkably, TGFβ1 levels can be affected by many other factors, such as growth factors and kinases, generating positive feed-forward loops necessary for sustained signaling that supports the EMT process [22,27].

We revealed that m^6^A methylation was critical to the EMT process of HeLa cells. We demonstrated that m^6^A methylation played at least three distinct roles in TGFβ1-induced EMT regulation—modulating TGFB1 mRNA decay and translation, TGFβ1 activation, and Snail expression—suggesting the multiple roles of m^6^A in cancer progression regulation. Notably, the regulation of METTL3 on TGFβ1-induced EMT might be cell type-dependent. The contribution of m^6^A in cancer metastasis in other types of cancer cells requires further investigation.

## Supplementary Materials

The following are available online at https://www.mdpi.com/2073-4409/9/2/296/s1, Figure S1: METTL3 regulates EMT in HeLa cells. (a) m^6^A/A ratio of total mRNA from control and Mettl3^Mut/−^ cells were determined by LC–MS/MS; (b) Mettl3^Mut/−^ cells transiently overexpressed METTL3 and then incubated with 10 ng/mL TGFβ1 for indicated times. The wound healing of cells was recorded (left) and quantitatively analyzed (right); scale bar, 100µm; (c) Mettl3^Mut/−^ cells transiently overexpressed METTL3 and then incubated with 10 ng/mL TGFβ1 and cells were allowed to invade for 24 h. Invaded cells were tested by CytoSelect™ 24-well Cell Invasion assay kits (8 µm, colorimetric format; left) and quantitatively analyzed (right); (d) Mettl3^Mut/−^ cells transiently overexpressed METTL3 and then incubated with or without 10 ng/mL TGFβ1 for 48 h. Protein levels of FN, E-Cad and METTL3 were measured by Western blot. Figure S2: Quality control for fractionation. Cytoplasmic (C) and nuclear (N) fractions were separated from control and Mettl3^Mut/−^ cells, respectively. Cytoplasmic marker GAPDH and nuclear marker H2A∙X were detected by Western blot. Figure S3: m^6^A methylation on both 5′UTR and CDS regions of TGFB1 mRNA control its translation efficiency. (a) Sequence of TGFB1 mRNA 5′UTR and CDS region (XM_011527242.2). TGFB1 5′UTR region is marked in blue. Mutation sites of potential m6A motifs were highlighted in red (mutation site: GGAC to GGCC); (b) WT-5′UTR or Mutant-5′UTR reporter was co-transfected with TK-Rluc reporter in Mettl3^Mut/−^ cells for 48 h. Dual-luciferase assay was performed to measure F-Luc production, which was normalized to R-Luc levels; (c) WT-CDS or Mutant-CDS reporter was transfected in control or Mettl3^Mut/−^ cells as indicated for 48 h. Expression levels of exogenous TGFβ1 (His) were measured by Western blot. Figure S4: Protein stability and secretion of TGFβ1 are modulated by METTL3. (a) Control and Mettl3^Mut/−^ cells were incubated with 10 ng/mL TGFβ1 for 48 h. 100 µg/mL cycloheximide (CHX) was added into cells for indicated times. Protein levels of mature TGFβ1 and TGFβ1 dimer were measured by Western blot (left). Band intensities were analyzed by ImageJ and listed at the bottom of target bands. Mature TGFβ1 levels were quantitatively analyzed (right); (b) Empty vector (Mock) and HA-LTBP1 vector were transiently overexpressed in Control and Mettl3^Mut/−^ cells. Expression levels of exogenous LTBP1 were detected by Western blot. Figure S5: m^6^A peaks of LTBP1, LTBP2, LTBP3 in HeLa cells. m^6^A peak distributions of LTBP1, LTBP2 and LTBP3 mRNA from m^6^A RIP-seq data (Accession code GSE112795). m^6^A abundance of mRNA was listed on the right. Table S1: Primers for mutagenesis.
