# Supplementary material for: N6-Methyladenosine Regulates the Expression and Secretion of TGFβ1 to Affect the Epithelial–Mesenchymal Transition of Cancer Cells
Source: Cells. 2020 Jan 25;9(2):296. doi: 10.3390/cells9020296 (PMC7072279; doi:10.3390/cells9020296)
Supplement: Supplementary file 1 [file cells-09-00296-s001.pdf]

Table S1. Primers for mutagenesis.

Name	Primers
pGL3-basic-Mut-5′UTR	Forward – cgcccttctccctgaggccctcagctttccctcgag
	Reverse – ctcgagggaaagctgagggcctcagggagaagggcg
pcDNA3-Mut-CDS	Forward – gccggccggccgcgggcctatccacctgcaagac
	Reverse – gtcttgcaggtggataggcccgcggccggccggc

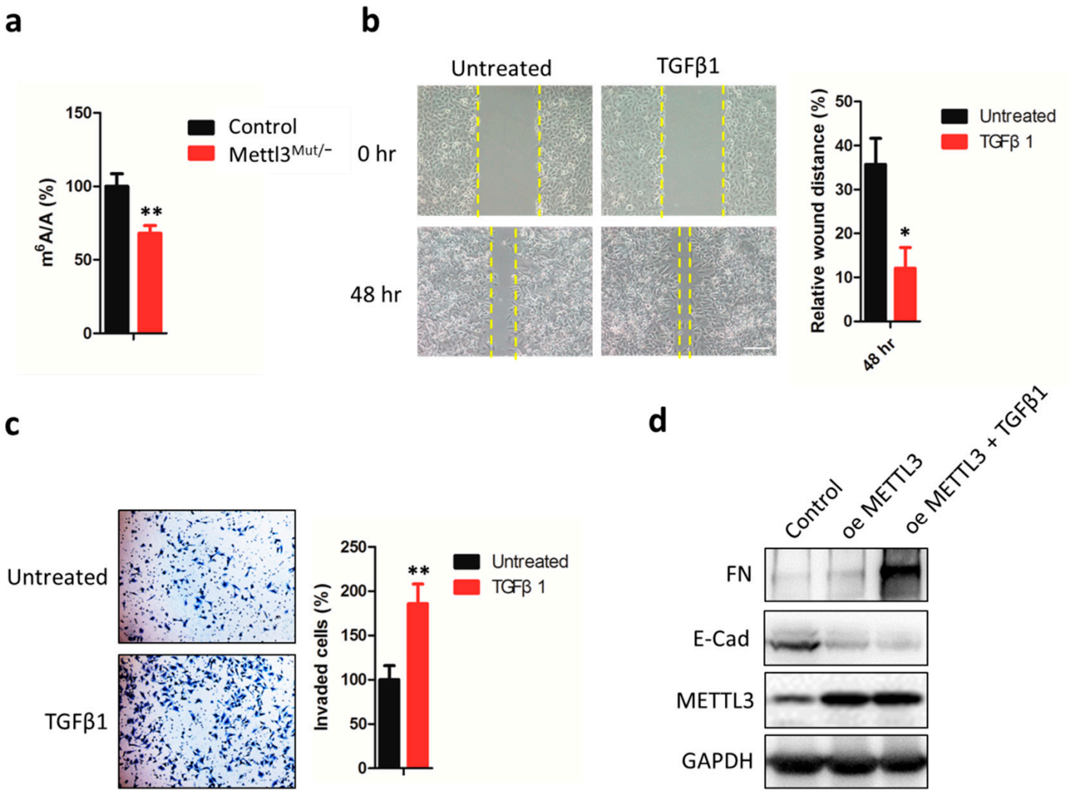

Figure S1. METTL3 regulates EMT in HeLa cells. (**a**) m^{6}A/A ratio of total mRNA from control and Mettl3$^{Mut/-}$ cells were determined by LC–MS/MS; (**b**) Mettl3$^{Mut/-}$ cells transiently overexpressed METTL3 and then incubated with 10 ng/mL TGFβ1 for indicated times. The wound healing of cells was recorded (*left*) and quantitatively analyzed (*right*); scale bar, 100μm; (**c**) Mettl3$^{Mut/-}$ cells transiently overexpressed METTL3 and then incubated with 10 ng/mL TGFβ1 and cells were allowed to invade for 24 h. Invaded cells were tested by CytoSelect™ 24-well Cell Invasion assay kits (8 μm, colorimetric format; *left*) and quantitatively analyzed (*right*); (**d**) Mettl3$^{Mut/-}$ cells transiently overexpressed METTL3 and then incubated with or without 10 ng/mL TGFβ1 for 48 h. Protein levels of FN, E-Cad and METTL3 were measured by Western blot.

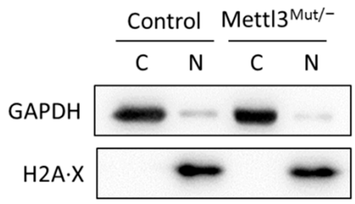

Figure S2. Quality control for fractionation. Cytoplasmic (C) and nuclear (N) fractions were separated from control and Mettl3$^{Mut/–}$ cells, respectively. Cytoplasmic marker GAPDH and nuclear marker H2A·X were detected by Western blot.

a

TATTTCCGTGGGATACTGAGACACCCCCGGTCCAAGCCTCCCCTCCACCACTGCGCCCTTCTCCCTGAGGACCTCAGCTTTCC
CTCGAGGCCCTCCTACCTTTTGCCGGGAGACCCCCAGCCCCTGCAGGGGCGGGGCCTCCCCACCACACCAGCCCTGTTCGC
GCTCTCGGCAGTGCCGGGGGGCGCCGCCTCCCCCATGCCGCCCTCCGGGCTGCGGCTGCTGCCGCTGCTGCTACCGCTGCT
GTGGCTACTGGTGCTGACGCCTGGCCGGCCGGCCGCGGGACTATCCACCTGCAAGACTATCGACATGGAGCTGGTGAAGC
GGAAGCGCATCGAGGCCATCCGCGGCCAGATCCTGTCCAAGCTGCGGCTCGCCAGCCCCCCGAGCCAGGGGGAGGTGCC
GCCCGGCCCGCTGCCCGAGGCCGTGCTCGCCCTGTACAACAGCACCCGCGACCGGGTGGCCGGGGAGAGTGCAGAACCG
GAGCCCGAGCCTGAGGCCGACTACTACGCCAAGGAGGTCACCCGCGTGCTAATGGTGGAAACCCACAACGAAATCTATGAC
AAGTTCAAGCAGAGTACACACAGCATATATATGTTCTTCAACACATCAGAGCTCCGAGAAGCGGTACCTGAACCCGTGTTGCT
CTCCCGGGCAGAGCTGCGTCTGCTGAGGCTCAAGTTAAAAGTGGAGCAGCACGTGGAGCTGTACCAGAAATACAGCAACA
ATTCCTGGCGATACCTCAGCAACCGGCTGCTGGCACCCAGCGACTCGCCAGAGTGGTTATCTTTTGATGTCACCGGAGTTGT
GCGGCAGTGGTTGAGCCGTGGAGGGGAAATTGAGGGCTTTCGCCTTAGCGCCCACTGCTCCTGTGACAGCAGGGATAACA
CACTGCAAGTGGACATCAACGCAGGGTTCACTACCGGCCGCCGAGGTGACCTGGCCACCATTCATGGCATGAACCGGCCTT
TCCTGCTTCTCATGGCCACCCCGCTGGAGAGGGCCCAGCATCTGCAAAGCTCCCGGCACCGCCGAGCCCTGGACACCAACT
ATTGCTTCAGCTCCACGGAGAAGAACTGCTGCGTGCGGCAGCTGTACATTGACTTCCGCAAGGACCTCGGCTGGAAGTGGA
TCCACGAGCCCAAGGGCTACCATGCCAACTTCTGCCTCGGGCCCTGCCCCTACATTTGGAGCCTGGACACGCAGTACAGCAA
GGTCCTGGCCCTGTACAACCAGCATAACCCGGGCGCCTCGGCGGCGCCGTGCTGCGTGCCGCAGGCGCTGGAGCCGCTGC
CCATCGTGTACTACGTGGGCCGCAAGCCCAAGGTGGAGCAGCTGTCCAACATGATCGTGCGCTCCTGCAAGTGCAGCTGA

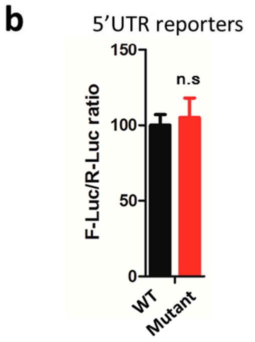

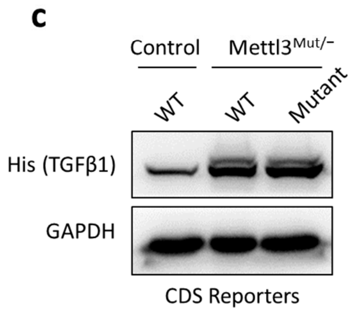

Figure S3. m^6A methylation on both 5′UTR and CDS regions of TGFB1 mRNA control its translation efficiency. (**a**) Sequence of TGFB1 mRNA 5′UTR and CDS region (XM_011527242.2). TGFB1 5′UTR region is marked in blue. Mutation sites of potential m^6A motifs were highlighted in red (mutation site: GGAC to GGCC); (**b**) WT-5′UTR or Mutant-5′UTR reporter was co-transfected with TK-Rluc reporter in Mettl3$^{Mut/-}$ cells for 48 h. Dual-luciferase assay was performed to measure F-Luc production, which was normalized to R-Luc levels; (**c**) WT-CDS or Mutant-CDS reporter was transfected in control or Mettl3$^{Mut/-}$ cells as indicated for 48hr. Expression levels of exogenous TGFβ1 (His) were measured by Western blot.

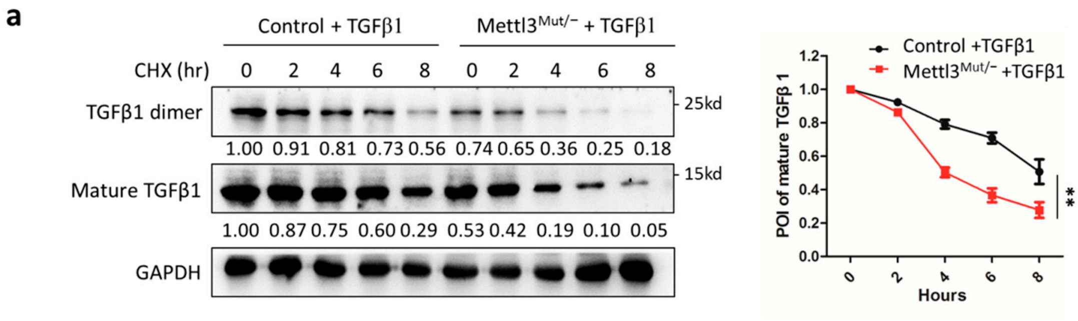

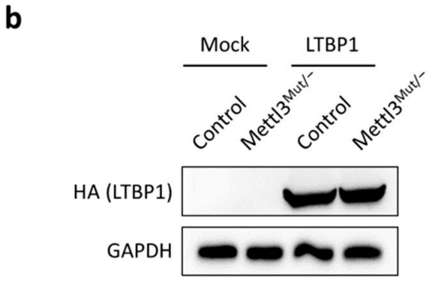

Figure S4. Protein stability and secretion of TGFβ1 are modulated by METTL3. (**a**) Control and Mettl3$^{Mut/-}$ cells were incubated with 10 ng/mL TGFβ1 for 48hr. 100μg/ml cycloheximide (CHX) was added into cells for indicated times. Protein levels of mature TGFβ1 and TGFβ1 dimer were measured by Western blot (*left*). Band intensities were analyzed by ImageJ and listed at the bottom of target bands. Mature TGFβ1 levels were quantitatively analyzed (*right*); (**b**) Empty vector (Mock) and HA-LTBP1 vector were transiently overexpressed in control and Mettl3$^{Mut/-}$ cells. Expression levels of exogenous LTBP1 were detected by Western blot.

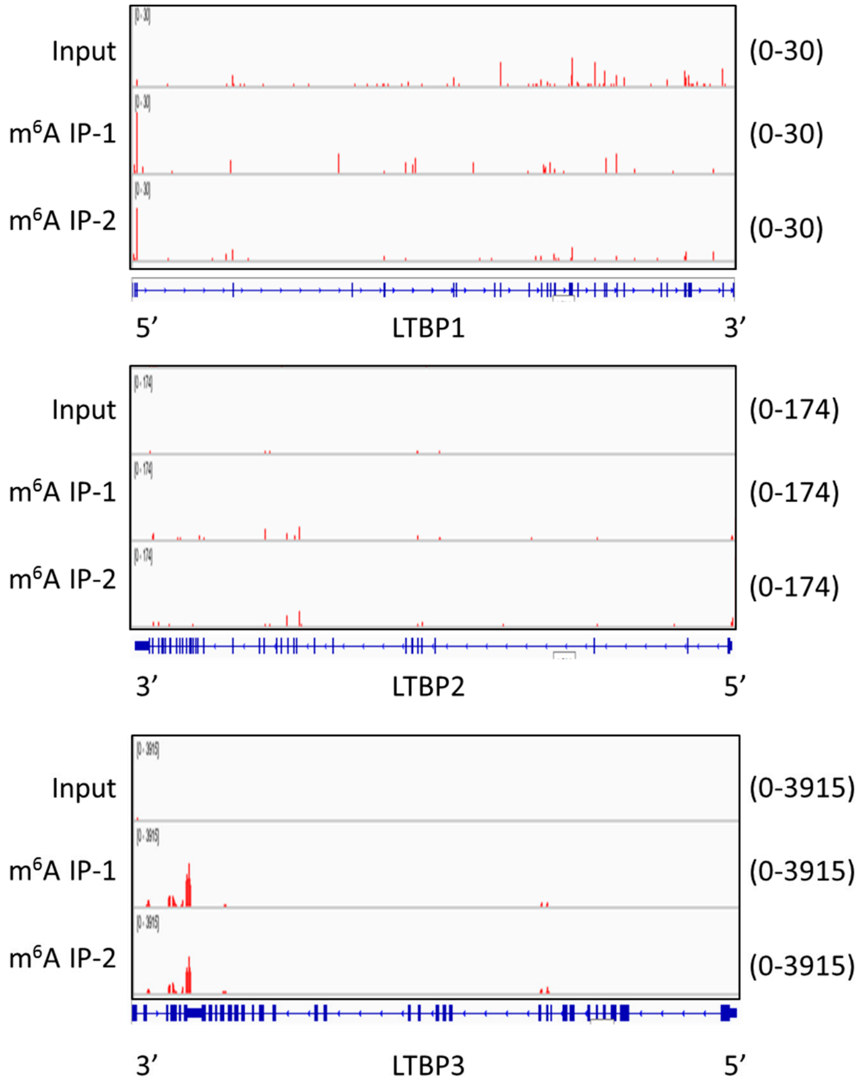

Figure S5. m^{6}A peaks of LTBP1, LTBP2, LTBP3 in HeLa cells. m^{6}A peak distributions of LTBP1, LTBP2 and LTBP3 mRNA from m^{6}A RIP-seq data (Accession code GSE112795). m^{6}A abundance of mRNA was listed on the right.
